# A functional polymorphism in *IFNAR1* gene is associated with susceptibility and severity of HFMD with EV71 infection

**DOI:** 10.1038/srep18541

**Published:** 2015-12-18

**Authors:** Rongrong Zou, Guoliang Zhang, Shaoyuan Li, Wenfei Wang, Jing Yuan, Jianming Li, Yanrong Wang, Yimin Lin, Yong Deng, Boping Zhou, George Fu Gao, Yingxia Liu

**Affiliations:** 1 Department of Infectious Diseases, Shenzhen Third People’s Hospital, University of South China, Shenzhen, 518112, China; 2 Guangdong Key Laboratory of Infectious Diseases, Shenzhen Third People’s Hospital, Guangdong Medical College, Shenzhen, 518112, China; 3 Shenzhen Key Laboratory of Pathogen and Immunity, Shenzhen Third People’s Hospital, Shenzhen, 518112, China; 4 Center for Molecular Virology, CAS Key Laboratory of Pathogenic Microbiology and Immunology, Institute of Microbiology, Chinese Academy of Sciences, 100101, Beijing.

## Abstract

Enterovirus 71 (EV71), one of the major pathogens of Hand, foot and mouth disease (HFMD), results in millions of infections and hundreds of deaths each year in Southeast Asia. Biased infection and variable clinical manifestations of EV71 HFMD indicated that host genetic background played an important role in the occurrence and development of the disease. We identified the mRNA profiles of EV71 HFMD patients, which type I interferon (IFN) pathway related genes were down-regulated. Four single nucleotide polymorphisms (SNPs) of type I IFN receptor 1 (IFNAR1) were chosen to analyze their relationships to EV71 infection. We found that genotype GG of promoter variant rs2843710 was associated with the susceptibility and severity to EV71 HFMD. In addition, we assessed the regulatory effects of rs2843710 to IFN stimulated genes (ISGs), and found that the expressions of IFNAR1, OAS1 and MX1 were significantly lower in patients with rs2843710 genotype GG. And rs2843710 allele G showed weaker transcriptional activity compared with allele C. Our study indicated that rs2843710 of IFNAR1 was associated with the susceptibility and severity of EV71 HFMD in Chinese Han populations, acting as a functional polymorphism by regulating ISGs expression, such as OAS1 and MX1.

Hand, foot and mouth disease (HFMD) is a common pediatric infectious diseases, it is caused by picornaviridae family member enterovirus, mainly caused by coxsackie virus A16 (CA16) and enterovirus 71 (EV71), which characterized by fever, oral mucosa herpes, and rash on the hands, foot, and buttocks. It usually affects those less than 5 years old, in particular under 3 years^1^,^2^,^3^. Although most HFMD patients have good prognosis, there are some patients with severe neurological complications such as aseptic meningitis, encephalitis, brain stem encephalitis, neurogenic pulmonary edema, and hemorrhage^4^,^5^, with a high mortality^6^. The number of HFMD had a nearly half millions and killed 126 people in 2008^7^,^8^. HFMD caused more than 1.6 million infectors and 509 deaths in 2011^9^, most victims were infectors of EV71^3^. Children with EV71 infection have a higher frequency of central nervous system complications. In recent 20 years, EV71 is mainly popular in Southeast Asia^4^,^10^,^11^, the national within the scope of the outbreak began in 2008 in China, and spread rapidly from Anhui to the other provinces^7^,^12^,^13^. Up to now, there are no effective vaccines and antiviral drugs to prevent or treat EV71 infection, so it is necessary to identify the susceptible factors or the warning signs to predict the disease progression.

HFMD is mainly popular in Southeast Asia, especially in China^4^,^10^,^11^,^12^, and mainly occurred in male patients^6^,^14^. Some patients may combine with severe central nervous system complications^11^,^15^,^16^. Even the same EV71 strain would lead to different clinical manifestations in different patients^17^. All together prompt that host genetic background plays an important role in the occurrence and development of EV71 HFMD. Polymorphisms of type I IFN signaling pathway genes like MX1 and OAS1 have been reported to link to the occurrence and development of HFMD^18^,^19^. Other variants of type II IFN related genes IFN-γ, IL-10 and IP-10, chemokines CCL2 and CXCL10 and eNOS also contributed to susceptibility or severity to EV71 infection^20^,^21^,^22^,^23^.

Type I IFN is the first line of host immune defense, and plays an important role in innate immune response and is an important cytokine to mediate host anti-viral response. Host recognized pathogen associated molecular patterns (PAMP) and activated type I IFN signal^24^. Type I IFN bound to IFNAR1/2^25^,^26^, and activate a range of antiviral IFN stimulated genes (ISGs), including protein kinase R (PKR), oligoadenylate synthestase (OAS) and interferon-induced GTP-binding protein Mx (MX)^27^,^28^,^29^,^30^. Both OAS and ds RNA dependent PKR modulate virus replication, and RNAase L and MX inhibit viral transcription^31^,^32^,^33^. On the other hand, EV71 could be survived through down-regulating IFNAR1 and JAK1 expression^34^,^35^, indicating that IFNAR1 plays an essential role in type I IFN signaling pathway against EV71 infection. It was reported that IFNAR1 gene polymorphisms were associated with many viruses infection, including HBV, HCV and HIV-1^36^,^37^,^38^, and variants of IFNAR1 downstream ISGs have been reported to link with the occurrence and development of HFMD^18^,^19^. However, the relationship between IFNAR1 polymorphisms and susceptibility of EV71 HFMD remains unknown.

In this study, we found that the expression of IFNAR1, IFNAR2, OAS1 and MX1 in PBMC reduced in patients with EV71 HFMD, particularly with severe symptoms. In the same time, we identified a genetic polymorphism rs2843710 C > G in the promoter of IFNAR1 gene was associated with the susceptibility and clinical phenotypes of EV71 HFMD, especially in male patients. In further, we found that rs2843710 allele G showed weaker transcriptional activity after EV71 infection. The expression of IFNAR1, OAS1 and MX1 reduced in patients with rs2843710 genotype GG compared with CC or CG. This may explain why rs2843710 allele G was associated with the susceptibility and severity of EV71 HFMD.

## Results

### EV71 HFMD was associated with the decrease of type I IFN related genes levels in PBMC

**Figure 1:**
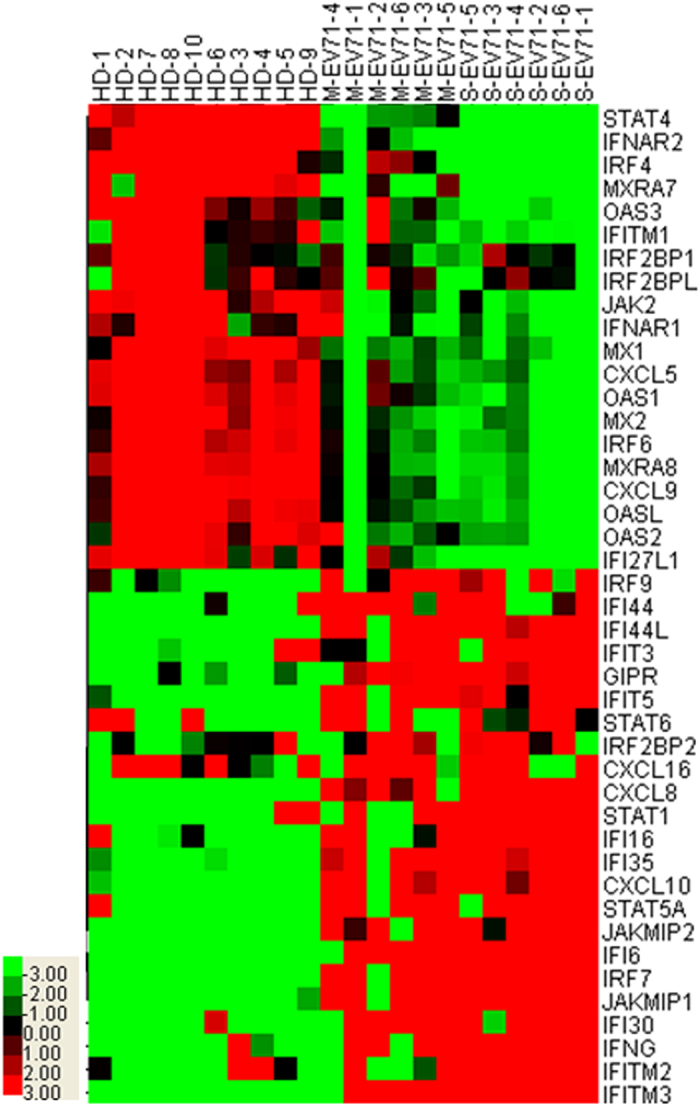
The mRNA expression profiles in PBMC from different populations. Genome-wide transcriptional analysis was performed in PBMC isolated from different groups (HD, n = 10; M-EV71, n = 6; S-EV71, n = 6). Raw data was performed using Illumina software, then delineated the false discovery rate (FDR) and normalized. *P* < 0.05 was regarded as statistical significant.

We performed genome-wide transcriptional analysis in PBMC isolated from healthy donors (HD, n=10), mild EV71 HFMD patients (M-EV71, n=6) and severe EV71 HFMD patients (S-EV71, n=6). Clustering analysis showed that the expression of 43 related genes was clearly different in HD, M-EV71 and S-EV71 groups, which could be divided into two gene clusters according to the different clustering model. The related genes of type I IFN signaling pathways had a similar expression patterns in the same cluster (Fig. 1).

**Figure 2:**
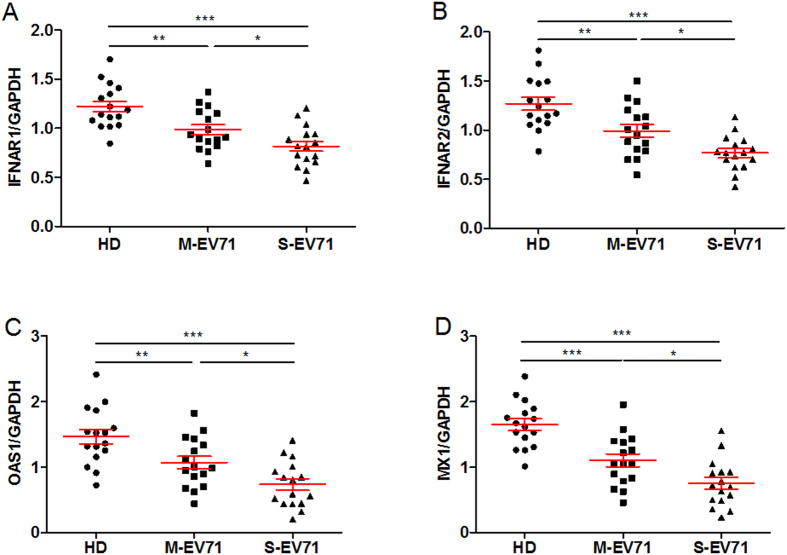
Validation of type I IFN related genes expression among different groups. (**A–D**) The IFNAR1 (**A**) and IFNAR2 (**B**) and OAS1 (**C**) and MX1 (**D**) mRNA levels were verified using SYBR Green qPCR in PBMCs from healthy controls, mild patients with EV71 infection and severe patients with EV71 infection (n = 16, respectively). The expression of mRNA was 2^−△△Ct^ relative to house keeping gene GAPDH. Differences between groups were compared with the ANOVA/Newman-Keuls multiple comparison test. *P < 0.05, **P < 0.01, ***P < 0.0001.

In particular, IFNAR1, IFNAR2, OAS1 and MX1 gene expression were significantly decreased in EV71 HFMD patients compared with HD. This difference was further validated by a conventional qPCR assay using another cohort (n = 16 for each group respectively, Fig. 2). Especially, the IFNAR1, IFNAR2, OAS1 and MX1 gene expression level was much lower in S-EV71 than that in M-EV71 (Fig. 2), suggesting the down-regulation of type I IFN related genes was associated with the severity of EV71 HFMD.

### Rs2843710 of IFNAR1 was associated with susceptibility and severity of EV71 HFMD

We selected 4 potential functional SNPs of IFNAR1 to assess their association with EV71 HFMD. Among four SNPs of IFNAR1, rs2843710 and rs1787572 located in the promoter region, rs113181057 in exon 11, and rs1012334 in intron 3. Four SNPs genotype distribution in cases and controls were coincident with Hardy-Weinberg equilibrium (*P* > 0.05). As shown in Table 1, the allele frequency of rs2843710 SNP was significantly different in HD (n = 573) and EV71 HFMD (n = 1196), and allele G showed higher risk to EV71 HFMD (OR = 1.34, 95% CI, 1.15–1.55; *P* = 0.0001). In genotype level, the patients with rs2843710 genotype GG showed increased risk of EV71 HFMD in the recessive model (OR = 1.33, 95% CI, 1.09-1.63; *P* = 0.005). Genotypes CC and CG showed decreased EV71 HFMD susceptibility compared with genotype GG using an additive model (OR = 0.49, 95%CI, 0.35–0.69; P < 0.0001; and OR = 0.58, 95%CI, 0.42–0.82, P = 0.002).

It was reported that EV71 HFMD mainly occurred in male patients^14^, so we further analyzed the association between rs2843710 and susceptibility in different sex. In male population (n = 738), the rs2843710 allele G frequency was significantly higher in cases than that in controls in multiplicative model (OR = 1.57, 95% CI, 1.29-1.90; *P* < 0.0001, Table 2). In genotype level, the patients with rs2843710 genotype GG increased risk of EV71 HFMD in the recessive model (OR = 1.63, 95% CI, 1.25 2.11; *P* = 0.0002, Table 2). In contrast, rs2843710 showed no association with susceptibility to EV71 HFMD in female population (n = 458).

To further clarify the relationship between rs2843710 and EV71 HFMD clinical manifestations, we compared different alleles’ frequencies in M-EV71 (n = 801) and S-EV71 (n = 395). The frequency of rs2843710 allele G was significantly higher in S-EV71 patients than that in M-EV71 patients, which suggested that the rs2843710 allele G was associated with the severity of EV71 HFMD (OR = 1.21, 95% CI, 1.02-1.44; *P* = 0.028, Table 3).

### Rs2843710 of IFNAR1 influenced IFNAR1, OAS1 and MX1 gene expression

**Figure 3:**
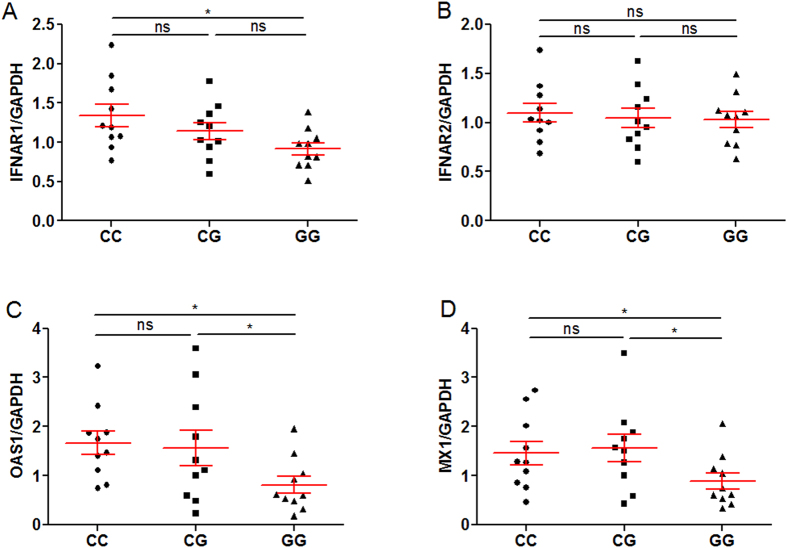
Rs2843710 of IFNAR1 influences mRNA expression of IFNAR1, OAS1 and MX1. (**A–D**) The IFNAR1 (**A**) and IFNAR2 (**B**) and OAS1 (**C**) and MX1 (**D**) mRNA levels were determined using SYBR Green qPCR in PBMCs from patients with different rs2843710 genotypes (CC, n = 10; CG, n = 10; GG, n = 10), and the expression of mRNA was 2^−△△Ct^ relative to house keeping gene GAPDH. Differences between groups were compared with the ANOVA/Newman-Keuls multiple comparison test. *P < 0.05; ns, not significant.

**Figure 4:**
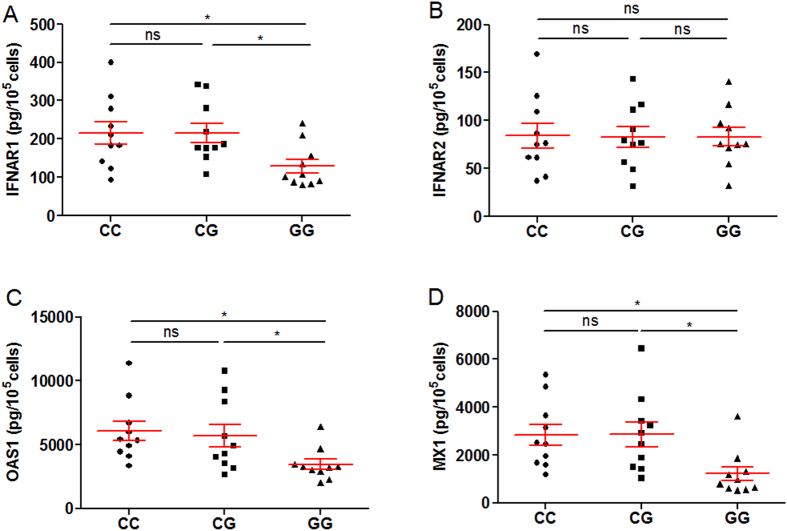
Rs2843710 of IFNAR1 affects IFNAR1, OAS1 and MX1 expression in protein level. (**A–D**) The IFNAR1 (**A**) and IFNAR2 (**B**) and OAS1 (**C**) and MX1 (**D**) protein levels were determined by ELISA assay in PBMCs from patients with different rs2843710 genotypes (CC, n = 10; CG, n = 10; GG, n = 10). Differences between groups were compared with the ANOVA/Newman-Keuls multiple comparison test. *P < 0.05; ns, not significant.

In order to explore the function of rs2843710, we detected the expression of IFNAR1, IFNAR2, OAS1 and MX1 in PBMC from patients with CC, CG and GG genotypes using qPCR and ELISA assay (n = 10, respectively). Interestingly, we found that the expression of IFNAR1, OAS1 and MX1 was much lower in genotype GG than that in genotypes CC or CG (Fig. 3A,C,D), while rs2843710 SNP did not affect the IFNAR2 gene expression (Fig. 3B). And lower protein expression of IFNAR1, OAS1 and MX1 was also observed in genotype GG carriers (Fig. 4A,C,D), while the protein expression of IFNAR2 showed no difference among all genotypes (Fig. 4B). Together, these results suggest that the rs2843710 genotype GG had specifically lower expression of IFNAR1 and down-stream genes.

### Rs2843710 of IFNAR1 affects IFNAR1 promoter transcriptional activity

**Figure 5:**
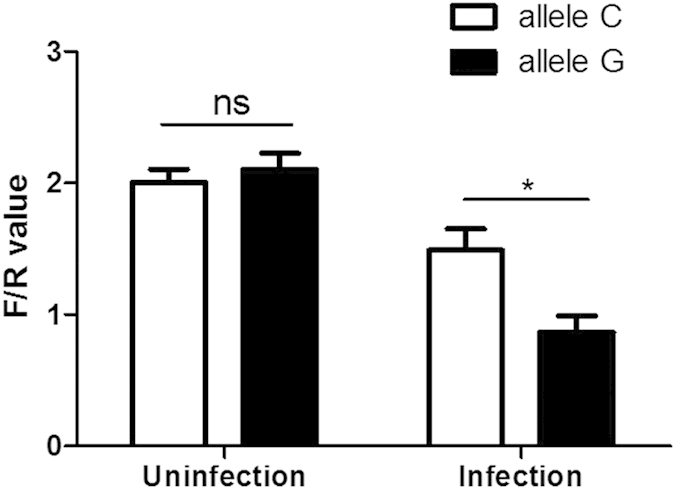
The rs2843710 SNP affects IFNAR1 promoter transcriptional activity. IFNAR1 promoter luciferase reporter plasmids carrying rs2843710 C or G allele were transfected into THP-1 cells, with or without EV71 infection. Luciferase activities of THP-1 cells were determined and normalized to Renillla luciferase activities. *P < 0.05; ns, not significant.

To evaluate the functional significance of promoter polymorphism, transcriptional activity of IFNAR1 promoter variant rs2843710 was tested by promoter luciferase reporter assay (Fig. 5). After EV71 infection, allele G of rs2843710 showed significantly weaker transcriptional activity than allele C, while no difference was observed between both alleles without infection. These results suggest that the transcriptional activity of IFNAR1 rs2843710 allele G was attenuated after EV71 infection.

## Discussion

In this study, we first illuminated the mRNA profiles of EV71 infection of HFMD by using mRNA Chip, and genes of type I IFN pathway were significantly decreased in EV71 HFMD patients. From more than 1700 volunteers and a Chinese case-control cohort, we identified a strong association between susceptibility and severity to hand, foot and mouth disease of EV71 infection and a genetic variation rs2843710 in the promoter region of IRFAR1. In contrast, other SNPs examined were not associated with disease susceptibility in the same populations. Moreover, allele G of rs2843710 showed weaker transcriptional activity compared with allele C in promoter luciferase reporter assay. The expression level of INFAR1 of rs2843710 genotype GG was lower than those with genotypes CC or CG, and coincided with the same expression pattern of INFAR1 downstream genes OAS1 and MX1, arguing that a tightly regulated type I IFN response to EV71 infection may have contributed to attenuate resistance to HFMD progression in this population.

Type I IFN comprised IFN-α, IFN-β, as well as some additional family members. It was the first line of host immune defense and directly responded to virus infection^39^,^40^. Type I IFN bound to its receptor (IFNAR), and activated a large amount of ISGs through JAK-STAT signal pathway^41^,^42^,^43^. Three ISGs systems present major antiviral activities, including PKR, OAS/Rnase L system and the MX GTPases^27^,^28^,^29^,^30^.

Type I IFN has protective effect against EV71 or CA16 infections. Type I IFN directly suppressed CA16 infection and controlled EV71 infection and replication^44^,^45^. IFN injection before EV71 infection could effectively protect mice, and the pre-infection effect was much stronger than post-injection^45^. All-trans-retinoic acid (ATRA) could protect cells apoptosis induced by EV71 infection and reduce the percentage of infected cells through enhancing the production of IFN-α^46^.

In contrast, EV71 could reduce the expression of IFNAR1. The 2A protein encoded by EV71 blocked type I IFN mediated STAT1, STAT2, Jak1 and Tyk2 phosphorylation through reducing IFNAR1 protein expression^34^. And 2A protein inhibited type I IFN responses by targeting the mitochondrial antiviral signaling (MAVS)^47^. The 3C protein of EV71 inhibited retinoid acid-inducible 1 gene mediated IRF3 activation and type I IFN responses^48^. And the 3C protein suppressed host immune response by blocking the type I IFN synthesis^49^. In addition, mir-146 expression increased after EV71 infection, and mir-146 promoted disease progression by inhibiting IFN production^34^.

HFMD is mainly popular in Southeast Asia and infects millions of children^4^,^10^,^11^,^12^, and the clinical manifestations are variable in different patients^17^, indicating that host genetic background played an important role in the occurrence and development of EV71 HFMD. Polymorphisms of type I IFN stimulated genes were associated with the susceptibility and clinical phenotypes of EV71 HFMD, such as −123 A and −88 T of MX1 obviously reduce the susceptibility of EV71, but individuals with the −123 A or −88 T had a higher MX1 mRNA levels in IFN-β stimulated PBMC compared with non-carriers^18^. A SNP rs10774671 of OAS1 contributed to the susceptibility and severity of CA16 HFMD and reduced IFN-γ expression level in severe HFMD patients^19^. Variants of type II IFN and other chemokines also contributed to EV71 HFMD. Interferon gamma induced proteins-10 (IP-10) gene −1596 T was the risk factor of EV71 infection^20^. Interleukin 10 (IL-10) and interferon gamma (IFN-γ) gene SNPs were associated with the susceptibility to EV71 HFMD encephalitis^21^. The −2518 G of CCL2, -201 A of CXCL10, 781 C of IL8 and 894 T of eNOS were associated with the severity of EV71 infection^22^,^23^.

We have found that IFNAR1 promoter polymorphism rs2843710 was related to susceptibility and severity of EV71 HFMD. Rs2843710, described as −568 C/G before, have been shown to be associated with susceptibility to chronic HBV infection^50^. And allele G of rs2843710 was associated with falciparum malaria manifestation in the endemic region^51^. Other variants in the promoter region of INFAR1 also related to the susceptibility of viruses infection. Carriers of 3 polymorphisms −408 T, −3 T, −77 GT_L_ had higher risk to HBV infection^50^, and polymorphisms −408 C can influence the risk of developing depression of hepatitis C virus^52^. It was reported that allele change C > T at the −3 locus reduced the transcriptional activity of IFNAR1 promoter^53^.

Since rs2843710 has been predicted near the binding site of GA binding protein transcription factor (GABP), this variant may affect IFNAR1 transcription and expression^53^. In our study, without infection, rs2843710 seemed to have no apparent impact on the transcriptional activity of IFNAR1, which was consistent with the result Zhou *et al.* have reported before^50^. However, after EV71 infection, allele G of rs2843710 showed significantly weaker transcriptional activity compared with allele C, indicating that the difference of transcriptional activity between G and C allele might be triggered by EV71 infection. Lower transcriptional activity of rs2843710 allele G impaired IFNAR1 gene expression and resulted in down-regulation of ISGs including MX1 and OAS1. According to previous report, EV71 infection could inhibit IFNAR1 expression^34^. Our promoter luciferase assay results showed that EV71 infection might involve in regulating IFNAR1 transcriptional activity and influence IFNAR1 expression. The role which EV71 played in IFNAR1 transcription was needed to be further research.

Most reports showed that EV71 infection was mainly occurred in male^6^,^14^,^54^. In our study, the frequency of rs2843710 susceptible genotype GG was significantly higher in male patients, while this frequency showed no difference between female cases and controls. Taken together, it indicated that higher susceptible of rs2843710 genotype GG in male might be one of the reasons why EV71 HFMD had higher prevalence in male population.

In conclusion, we have identified a functional polymorphism rs2843710 in IFNAR1 gene promoter region was associated with susceptibility and severity of HFMD with EV71 infection. After EV71 infection, allele G of rs2843710 attenuated IFNAR1 transcriptional activity and expression compared with allele C, resulting in the decrease of interferon stimulated genes including MX1 and OAS1. Lower expression of MX1 and OAS1 reduced inhibition effect to virus, causing higher risk to EV71 HFMD or severer progression and complications. Higher susceptible of rs2843710 genotype GG in male might contribute to higher EV71 HFMD prevalence in male population.

## Methods

### Study subjects and sample

All EV71 HFMD patients were recruited from department of infectious disease or pediatric in the Third People’s Hospital of Shenzhen in 2012–2014. Healthy donors (HD, n = 573) who underwent a health examination were recruited from Shenzhen during the same period, there was no history of HFMD for HD and serum EV71 IgG test showed negative (Table 4). The criteria of HFMD diagnostics was referred to the ministry of health, 2010 version of the “hand, foot and mouth disease diagnosis and treatment guidelines”. EV71 HFMD patients were divided into mild and severe EV71 HFMD patients (M-EV71, n = 801; S-EV71, n = 395). S-EV71 presented with meningitis, encephalitis, encephalomyelitis, pulmonary edema, myocarditis, circulation problems, and ruled out merger other common infectious diseases, such as measles, cytomegalovirus infection, mumps, etc; M-EV71 didn’t get central nervous system complications and eliminated merger other common infectious diseases. We collected 3 ml peripheral blood from each volunteer using lithium heparin anticoagulant tube. 200 μl blood was cryopreserved −20 °C for genomic DNA extraction, and the remaining was for PBMC separation by density gradient centrifugation over Ficoll-Hypaque as described before^55^. EV71 RNA was assessed by Enterovirus type 71 nucleic acid detection kit (Da An Gene Co., Ltd., Guangzhou, China), and each patient was positive. All clinical procedures followed the protocols and were carried out in accordance with the approved guidelines of the Ethical Committee of the Third People’s Hospital of Shenzhen, under approval number TPHSZ2012001. All participants provided their written consents for the current study.

### DNA and RNA Extraction

Blood genomic DNA extraction was isolated from the 200 μL whole blood using QIAamp Mini kit (Qiagen, Germany) according to the kit instructions. The quality and concentration of DNA was detected with ultramicro spectrophotometer (Agilent companies in the United States). The concentration of DNA was diluted into 5 ng/μl. RNA was extracted from 1 × 10^6^ PBMC using RNeasy Mini Kit (Qiagen, Germany) according to the kit instructions. The quality and concentration of RNA was detected using ultramicro spectrophotometer (Agilent, USA). DNA and RNA was stored −80 °C for long-term preservation.

### Microarray data analysis

RNA specimens was reverse transcribed to synthesize cDNA, then inspecting the quality of cDNA based on Illumina instructions, sample hybridization, labeling, and scanned on the iScan systems. For microarray assays, we used the HumanHT-12 v4 Expression BeadChip with 47 000 probes representing 25000 annotated genes, which coverage of well-characterized genes, gene candidates, and splice variants (Illumina, San Diego, CA, USA). Raw data generated from Illumina HT-12 was performed using the Illumina software, then delineate the false discovery rate (FDR) and normalized. *P* < 0.05 was regarded as Statistical significant.

### Measurement of genes expression with SYBR Green qPCR assay

The mRNA expression was validated with SYBR Green qPCR assay and GAPDH was considered as a house keeping gene. RNA was reversed transcribed into cDNA using PrimeScriptTM RT reagent Kit (Takara companies, Japan). The qPCR reaction system was as follows: SYBR Premix Ex Taq II 10.0 μL, PCR Forward Primer (10 μM) 0.8 μL, PCR Reverse Primer (10 μM) 0.8 μL, ROX Reference Dye II (50×) 0.4 μL, cDNA solution 1.0 μL, RNase free H_2_O 7.0 μL. Reaction conditions as follows: 95 °C, 30 s; 95 °C, 5 s, 60 °C, 34 s, 40 cycles. The data were expressed as mRNA copy number relative to the house keeping gene GAPDH.

### SNP Selection and Genotyping

SNPs were selected according to the SNP in HapMap database information, with focusing on their potential regulatory roles, such as transcription binding sites in the promoter region, microRNA target sites in the 3′ untranslated region (UTR), protein phosphorylation sites in the extrons and other putative regulation sites. SNPs were genotyped by time of flight mass spectrometry technology (Sequenom, San Diego. CA). The genotypes were determined by a Homogenous Mass EXTEND assay. Data analysis was performed by using MassARRAY typer software 4.0.

### Measurement of IFNAR1, IFNAR2, OAS1 and MX1 expression in PBMCs with ELISA

The whole PBMCs from different genotypes patients were lysed by diluting 1:20 in hypotonic buffer as described previously^56^, Diluted specimens were frozen and stored at −80 °C. The levels of INFAR1 and IFNAR2 were determined using commercially available ELISA kits (PBL Assay Science, Piscataway, NJ), and the measurement of OAS1 and MX1 was also determined using a sandwich enzyme immunoassay kit BioVendor, Sweden) followed the manufacturer’s instructions.

### Plasmid Constructs and Mutagenesis

The human IFNAR1 promoter (−1282 to +49) was amplified by PCR and inserted into pGL3-Basic vector (Promega, Madison, WI) upstream of the firefly luciferase coding region at Xho I and Hind III site. The primers used to PCR genomic DNA, were 5′-CATCTCGAGGAGATGGAATATAGAGATGGAATAG-3′ (forward, underlined letters showed Xho I site) and 5′-CATAAGCTTACGCGCTGCCCCTCTTAGCTCTCAC-3′ (reverse, underlined letters showed Hind III site). The pGL3-rs2843710-C vector was mutated into pGL3-rs2843710-G by site-directed mutagenesis using QuickChang II Site-Directed Mutagenesis Kit (Agilent Technologies, Santa Clara, CA). The primers used for mutation were 5’-TCTGCCCCGCTCTCG**G**TCTGCACACAGCAAC-3′ (forward) and 5′- GTTGCTGTGTGCAGA**C**CGAGAGCGGGGCAGA-3′ (reverse), in which the bold letter showed the altered nucleotide.

### Transient transfection and Reporter Assays

A human monocytic cell line (THP-1) was grown at 37 °C in 5% CO2 in RPMI 1640 medium with 10% FBS. To make macrophages, THP-1 cells were incubated with PMA (20 ng/ml) in 24-well plates for 24 hrs, and then washed with fresh media before transfection. A total of 0.5 ug plasmid DNA including 0.4 ug of either rs2843710-C or rs2843710-G reporter vector, and 0.1 ug of pRL-TK control vector, were co-transfected into cells using jetPEI reagents according to the manufacture’s instruction (Polyplus). 24 hrs later, the cells was infected with EV71 at a MOI of 10 for 2 hrs at 37 °C. Unbound viruses were removed by washing them with medium. The EV71 strain (GenBank: FJ607335.1) was originally isolated from the throat swabs of a HFMD patients and amplified in Rhabdomyosarcoma (RD) cell. Infected cells were then harvested and detected the Firefly and Renilla luciferase activities with Dual-Glo luciferase reporter assay system (Promega) following 48 hrs of infection. Promoter activity was measured as the ratio between Firefly and Renilla luciferase. All experiments were performed at least twice, with each transfection in triplicate.

### Statistical Analysis

All SNPs investigated in this study were tested for Hardy-Weinberg equilibrium. The pearson χ^2^ test was used to evaluate distribution of allele frequencies in cases and controls. The unconditional logistic regression adjusted by gender and age were performed to calculate the Odd ratios (ORs), 95% confidence intervals (CIs) and corresponding P values under four alternative models (multiplicative, additive, dominant and recessive). The one-way analysis of variance (ANOVA)/Newman-Keuls multiple comparison test was used for statistical analyses to compare the differences among multiple groups. Differences were considered significant for *P* < 0.05. Statistical analysis of experimental data was performed using GraphPad Prism software version 5.0 (GraphPad Software, La Jolla, CA).

## Additional Information

**How to cite this article**: Zou, R. *et al.* A functional polymorphism in *IFNAR1* gene is associated with susceptibility and severity of HFMD with EV71 infection. *Sci. Rep.* **5**, 18541; doi: 10.1038/srep18541 (2015).

## Figures and Tables

**Table 1:** The association between IFNAR1 gene SNPs and EV71 HFMD susceptibility.

SNP ID	Genotype	HD	EV71	Multiplicative		Additive		Dominant		Recessive	
		No (%)	No (%)	P Value	OR (95% CI)	P Value	OR (95% CI)	P Value	OR (95% CI)	P Value	OR (95% CI)
rs2843710	CC	251 (43.8)	442 (37.0)	0.0001	1.34(1.15–1.55)	<0.0001	0.49(0.35–0.69)	0.0002	0.54(0.39–0.74)	0.005	1.33(1.09–1.63)
	CG	269 (46.9)	565 (47.2)			0.002	0.58(0.42-0.82)				
	GG	53 (9.3)	189 (15.8)			Ref.	Ref.				
rs1787572	GG	196 (34.2)	466 (39.0)	0.2019	0.91(0.79–1.05)	0.4725	0.89(0.66–1.21)	0.8764	1.02(0.78–1.35)	0.0530	0.81(0.66–1.00)
	GT	290 (50.6)	545 (45.6)			0.4067	1.13(0.85–1.52)				
	TT	87 (15.2)	185 (15.4)			Ref.	Ref.				
rs113181057	TT	573(100.0)	1196(100.0)	—	—	—	—	—	—	—	—
	TC	0 (0.0)	0 (0.0)			—	—				
	CC	0 (0.0)	0 (0.0)			Ref.	Ref.				
rs1012334	AA	195 (34.0)	410 (34.3)	0.2813	0.92(0.80–1.07)	0.1516	0.81(0.60–1.08)	0.0607	0.78(0.60–1.01)	0.9175	0.99(0.80–1.22)
	AT	268 (46.8)	599 (50.1)			0.0517	0.76(0.58–1.00)				
	TT	110 (19.2)	187 (15.6)			Ref.	Ref.				

Note: Hardy-Weinberg equilibrium P values of 4 SNPs (in the order of rs2843710, rs17875752, rs113181057, rs1012334) were 0.11, 0.23, 0.00, and 0.30 in HD, and were 0.71, 0.22, 0.00, and 0.19 in EV71. Abbreviations: HD, healthy donors; EV71, EV71 HFMD; CI, confidence interval; OR, odds ratio.

**Table 2:** The association between the rs2843710 SNP and EV71 HFMD susceptibility stratified by sex.

Sex	Genotype	HD	EV71	Multiplicative		Additive		Dominant		Recessive	
		No (%)	No (%)	P Value	OR (95% CI)	P Value	OR (95% CI)	P Value	OR (95% CI)	P Value	OR (95% CI)
Male	CC	164 (47.8)	266 (36.0)	<0.0001	1.57(1.29–1.90)	< 0.0001	0.35(0.22–0.57)	0.0001	0.43(0.27–0.67)	0.0002	1.63(1.25–2.11)
	CG	153 (44.6)	353 (47.8)			0.0034	0.50(0.32–0.80)				
	GG	26 (7.6)	119 (16.2)			Ref.	Ref.				
Female	CC	87 (37.8)	176 (38.4)	0.5957	1.07(0.84–1.34)	0.3426	0.84(0.51–1.39)	0.2075	0.79(0.50–1.26)	0.8782	0.97(0.70–1.35)
	CG	116 (50.4)	212 (46.3)			0.1679	0.76(0.46–1.23)				
	GG	27 (11.8)	70 (15.3)			Ref.	Ref.				

Note: Hardy-Weinberg equilibrium P values of rs2843710 in males and females were 0.23, 0.99, in HD, and were 0.21, 0.54 in EV71.

Abbreviations: HD, healthy donors; EV71, EV71 HFMD; CI, confidence interval; OR, odds ratio.

**Table 3:** The association between rs2843710 SNP and severity of EV71 HFMD.

SNP ID	Genotype	HD	M-EV71	S-EV71	M-EV71 Vs HD		S-EV71 Vs HD		S-EV71 Vs M-EV71	
		No (%)	No (%)	No (%)	P Value	OR (95% CI)	P Value	OR (95% CI)	P Value	OR (95% CI)
**rs2843710**	CC	251 (43.8)	310 (38.7)	132 (33.4)	0.0053	1.25(1.07–1.47)	< 0.0001	1.52(1.26–1.84)	0.0289	1.21(1.02–1.44)
	CG	269 (46.9)	375 (46.8)	190 (48.1)						
	GG	53 (9.3)	116 (14.5)	73 (18.5)						

Note: Hardy-Weinberg equilibrium P values of rs2843710 were 0.11 in HD, and were 0.88 in M-EV71, and were 0.75 in S-EV71. Abbreviations: HD, healthy donors; M-EV71, mild EV71 HFMD; S-EV71, severe EV71 HFMD; CI, confidence interval; OR, odds ratio.

**Table 4:** Characteristics of patients with HFMD with EV71 infection and healthy donors.

Cohort	Subgroup	No.	Age, months (mean ± SD)	Sex (Male:Female)
Healthy donors	—	573	25.37 ± 11.00	343:230
EV71	M-EV71	801	25.01 ± 17.42	482:319
	S-EV71	395	24.71 ± 14.28	256:139
